# Gram-Negative Bacterial Overgrowth Predominated by: Potential Association with Intestinal Barrier Impairment and Inflammatory Diarrhea in Cats

**DOI:** 10.3390/vetsci13090976

**Published:** 2026-09-17

**Authors:** Jing Wang, Xinhui Zhang, Xinran Song, Yan He, Wuying Lang, Xinyu Zhang

**Affiliations:** 1 College of Modern Agriculture and Bioengineering, Shangluo University, Shangluo 726000, China; 2 College of Agriculture, Chifeng University, Chifeng 024000, China; 3 State Key Laboratory of Animal Nutrition and Feeding, College of Animal Science and Technology, China Agricultural University, Beijing 100193, China

**Keywords:** fecal microbiota transplantation (FMT), intestinal barrier, inflammatory cytokines, *Escherichia*–*Shigella*

## Abstract

Background: Feline diarrhea is one of the most common gastrointestinal disorders encountered in veterinary clinics. Dysbiosis of Gram-negative bacteria is widely presumed to be a key cause of this disease, yet adequate in vivo evidence demonstrating its roles in provoking inflammation and impairing intestinal barrier integrity is still lacking. In this study, we investigated the effects of bacterial flora in cats with diarrhea on gut health in mice. Results: The results show the following: (1) Microorganisms in feces of diarrheal cats can cause weight loss in antibiotic-treated mice; the histopathological section of the colon showed that neutrophil aggregation occurred in the colon of mice in the fecal bacteria transplantation group; this indicates that gavage using fecal microbes from diarrheic cats could impair intestinal barrier integrity in antibiotic-treated recipient mice. (2) In addition, microbes in cats with diarrhea can induce inflammation in the colon and aggregation of macrophages, which may be caused by some Gram-negative bacteria in feces of cats with diarrhea. (3) The composition of the intestinal microbial community was analyzed by 16S rRNA gene sequencing, and it was found that the relative abundance of *Escherichia*–*Shigella* in feces of cats with diarrhea was significantly higher than that of normal cats. (4) Quantitative analysis of fecal samples revealed that diarrheic cats exhibited a significantly higher load of fecal Gram-negative bacteria compared with healthy cats, verifying that excessive colonization of Gram-negative bacteria may be highly correlated with feline diarrhea. Conclusion: In summary, fecal microbes from diarrheic cats are capable of triggering intestinal inflammation and barrier damage in antibiotic-treated mice. Excessive proliferation of Gram-negative bacteria, mainly *Escherichia*–*Shigella* species, may be highly associated with feline diarrhea.

## Simple Summary

Feline diarrhea is a common gastrointestinal disease in veterinary clinics. It is hypothesized that dysbiosis of Gram-negative bacteria serves as a critical predisposing factor for this disease; however, in vivo evidence confirming that such microbiota triggers intestinal inflammation and disrupts intestinal barrier function remains scarce. In this study, fecal microbiota from diarrheic cats was transplanted into antibiotic-pretreated mice to investigate its impacts on host intestinal health. The results demonstrated that fecal microbes from diarrheic cats reduced body weight in mice, induced infiltration of neutrophils and macrophages in colonic tissues, and elicited intestinal inflammation together with intestinal barrier injury. 16S rRNA sequencing revealed that the relative abundance of *Escherichia–Shigella* in feces of diarrheic cats were markedly higher than that in healthy cats. Quantitative assays also verified a significantly increased load of fecal Gram-negative bacteria in diarrheic cats. Collectively, fecal microbiota from diarrheic cats is capable of inducing intestinal inflammation and barrier damage in mice. Overproliferation of Gram-negative bacteria, represented mainly by *Escherichia–Shigella*, is closely associated with the occurrence of feline diarrhea.

## 1. Introduction

Diarrhea is one of the most prevalent gastrointestinal disorders in feline veterinary practice. Feline diarrhea leads to inappetence, growth retardation, body weight loss and impaired immunity [1,2]. Chronic long-term diarrhea further triggers intestinal mucosal injury, digestive dysfunction, and even secondary systemic inflammation and infection, which may result in death in severe cases, bringing substantial troubles to companion cat husbandry and health management [3]. The pathogenic factors underlying pet diarrhea are complicated, mainly including parasitic infection, viral infection, dietary stress, bacterial infection and other causes. Clinically, mature vaccination protocols are available for the prevention of viral and parasitic diseases in companion animals; nevertheless, bacterial vaccines are poorly popularized and fail to provide effective immune protection [4]. Accordingly, bacterial infection has become the predominant trigger of pet diarrhea, particularly household cat diarrhea.

Household cats have limited activity ranges with minimal microbial exchange with the external environment, maintaining relatively stable intestinal flora. Such cats are therefore susceptible to dysbiosis triggered by mild external stimuli, ultimately developing diarrhea. Most cases of bacterial diarrhea in cats present with mild clinical manifestations, predominantly loose stools and transient watery feces, which are frequently overlooked by cat owners. This oversight allows diseased cats to carry pathogens persistently and act as potential pathogen reservoirs, posing notable public health risks [5,6]. For instance, *Escherichia coli* and *Shigella* shed in cat feces survive for up to 11 days at room temperature and 90 days under low-temperature conditions [7]. These pathogens are major causative strains of dysentery in immunocompromised human populations, creating prominent health threats to cat owners.

Intestinal dysbiosis, especially excessive proliferation of Gram-negative bacteria, is widely recognized as a pivotal contributor to feline diarrhea. Previous studies have identified significant discrepancies in intestinal microbiota between healthy and diarrheic cats [8]. Diarrheic cats exhibit reduced intestinal microbial diversity and disrupted flora structure, with marked enrichment of typical Gram-negative bacteria such as *Shigella* and *Escherichia coli* in feces; these taxa are hypothesized to serve as major potential pathogens responsible for bacterial diarrhea in cats [9,10]. Lipopolysaccharide (LPS) embedded in the cell wall of Gram-negative bacteria elicits robust inflammatory responses in the intestinal mucosa, recruits inflammatory cells including neutrophils and macrophages to infiltrate intestinal tissues, disrupts intestinal mucosal immune homeostasis, and eventually induces intestinal damage and diarrhea [11,12]. Current research on enteric pathogens of diarrheic cats mostly focuses on alterations in microbial community composition. Few studies have clarified whether abnormal fecal microbiota from diarrheic cats triggers inflammatory cell infiltration, modulates the expression of tight junction proteins, and further compromises intestinal barrier integrity in vivo [13,14].

Fecal microbiota transplantation (FMT) represents a classic approach for verifying causal relationships between intestinal flora and host diseases. This technique can directly and authentically characterize the pathogenic phenotypes of gut microbiota in vivo, overcoming the limitation that high-throughput sequencing alone only demonstrates correlative associations rather than causation [15,16]. To date, FMT has rarely been adopted to validate the pathogenicity of gut microbiota in feline diarrhea, resulting in a lack of direct in vivo evidence supporting the pathogenic role of dysregulated intestinal flora in diarrheic cats.

To address these research gaps, the present study enrolled healthy cats and cats with spontaneous diarrhea as research subjects. Gut microbiota sequencing was performed to characterize key differential pathogenic bacterial profiles in diarrheic cats. Combined with a mouse FMT model, we systematically verified the intestinal damaging effects of fecal microbiota from diarrheic cats across multiple dimensions, including individual phenotypic changes, histopathology, inflammatory cell infiltration, and intestinal tight junction protein expression. This study aims to elucidate the pathogenic mechanism by which disordered Gram-negative bacteria provoke intestinal inflammation and barrier damage, confirm the causal link between gut dysbiosis and feline diarrhea, and supplement theoretical evidence regarding the pathogenesis of bacterial diarrhea in domestic cats.

## 2. Materials and Methods

### 2.1. Experimental Animals and Design

We utilized six-week-old male C57BL/6J mice, sourced from Huafu Kang in Beijing, China. After a 7-day acclimation period in the animal housing facility, the mice were housed individually in ventilated cages, with a maximum of five mice per cage. They had ad libitum access to food and water, and were maintained in an environment with a temperature range of 18–23 °C, 40–60% humidity, and a 12 h light/dark cycle. The mice selected for the study were of similar body weight to ensure consistency. The experimental protocols were approved by the Institutional Animal Care and Use Committee of China Agricultural University. The mice received a 7-day pre-treatment with a combination of quadruple antibiotics to fecal microbiota transplantation (ampicillin 1 mg/mL, metronidazole 1 mg/mL, kanamycin sulfate 1 mg/mL, and vancomycin 0.5 mg/mL). The fecal samples were inoculated onto BHI solid medium and cultured under anaerobic conditions at 37 °C for 48 h and aerobic conditions at 37 °C for 24 h. If the number of bacteria cultivated was less than 10^3^ CFU/g and the reduction rate of the bacterial population was over 90%, this could be regarded as a significant indicator of the successful establishment of the antibiotic-treated mouse model [17]. It should be noted that this culture-based assay only detects culturable microbes and cannot represent the entire intestinal microbial community. After antibiotic treatment, the mice were randomly divided into two groups, with 12 replicates in each group, and 1 mouse in each replicate. Notably, an FMT group receiving fecal microbiota from healthy cats was not included in this study.

### 2.2. Fecal Microbiota Transplantation (FMT)

The animals were recruited from the Veterinary Teaching Hospital of China Agricultural University. In this study, 27 cats with diarrhea and 7 healthy cats were enrolled. All cats were housed indoors for at least 6 months prior to sample collection. Detailed individual information of all diarrheic animals is provided in Supplementary Table S1. During this period, all cats received regular deworming treatment and were vaccinated with the cat pentavalent vaccine. This was done to eliminate the risk of exposure to parasites or pathogens due to outdoor activities. Antibiotics, antifungal drugs, or immunosuppressants had not been used within the past 6 months. Daily feeding consisted mainly of commercial dry food or wet food. The fecal scores of the healthy cats were within the range of 2–3, which were considered normal feces, and there were no gastrointestinal symptoms such as diarrhea or vomiting in the past three months. The fecal scores of the cats with diarrhea were less than 1.5. During the fecal collection process, independent sterile fecal collection containers were used. The feces of normal cats are used to detect the composition of microorganisms. Fecal samples from each diarrheic cat were collected at identical weights and supplemented with an equal-volume microbial protectant. Each sample was homogenized, filtered, and processed within 2 h post-collection before being resuspended in physiological saline. The suspension was further filtered through gauze, and the filtrate was centrifuged at 1000× *g* for 3 min. Supernatants (containing gut microbiota) obtained from all 27 diarrheic cats were pooled by mixing equal volumes to generate a single composite donor inoculum, and this pooled inoculum was used for gavage of all mice in the FD group. The supernatant was subsequently preserved in glycerol and stored at −80 °C to strictly avoid repeated freezing and thawing. Mice that had been treated with antibiotics were administered 0.2 mL of the liquid via gavage once a day for a period of 15 days.

### 2.3. Sample Collection

Mice were euthanized by cervical dislocation, and their livers, spleens, and kidneys were harvested and weighed. The colon was also harvested and its length measured. After removing the contents, the colon was divided into two sections; one part was placed in 4% paraformaldehyde, and the remaining part was quickly stored in a −80 °C freezer.

### 2.4. RNA Extraction and Rt-Pcr

Total RNA was extracted using TRIzol reagent according to the manufacturer’s instructions, and RNA was reverse transcribed into cDNA using a reverse transcription kit following the instructions provided. RT-PCR reactions were performed according to the manufacturer’s protocol. Primers were designed based on the principles of PCR primer design, with *β-actin* used as the internal reference gene. All primers were purchased from Shanghai Sangon Biotechnology Co., Ltd., China, Shanghai, and detailed information is listed in Table 1.

### 2.5. Tissue Staining and Imaging

Intestinal tissue samples were fixed in 4% paraformaldehyde, paraffin-embedded, and serially sectioned at 5 μm thickness. For histological evaluation, routine hematoxylin and eosin (H&E) staining was performed according to standard protocols, and tissue micrographs were acquired under an optical microscope with 10× and 20× objective lenses. Intestinal mucosal damage was blindly evaluated using a semi-quantitative histological scoring system. For each sample, three non-serial sections were prepared, and five random, non-overlapping fields per section were analyzed for morphological assessment and scoring. Intestinal histological injury was evaluated using a blinded semi-quantitative scoring system. For each mouse, three non-serial intestinal sections were prepared, and five random, non-overlapping fields per section were selected for imaging. Four indicators including epithelial integrity, inflammatory cell infiltration, mucosal edema, and villus morphology were scored from 0 (no lesion) to 3 (severe lesion). The total score of each field was summed, and the average value of all fields was taken as the final histopathological score for each sample.

TUNEL staining was conducted using a commercial TUNEL assay kit (Solarbio, Beijing, China) following the manufacturer’s standard instructions to detect intestinal epithelial apoptosis. For immunofluorescence staining, paraffin sections were dewaxed and rehydrated, followed by antigen retrieval to expose masked antigenic sites. After blocking, sections were incubated with primary antibodies overnight at 4 °C. The primary antibodies used included rabbit anti-F4/80 (1:200, Solarbio, Beijing, China) and rabbit anti-CD11b (1:200, Solarbio, Beijing, China). After thorough washing, sections were incubated with Alexa Fluor 488-conjugated goat anti-rabbit IgG secondary antibody (1:500, Solarbio, Beijing, China) at room temperature. Nuclei were counterstained with DAPI for 5 min, and sections were mounted with fluorescent mounting medium. All immunofluorescence and TUNEL images were captured using a laser scanning confocal microscope (ECLIPSE Ti2, Nikon, Japan), with five random non-overlapping fields captured per section. All representative images shown in the figures were selected from the quantified and analyzed datasets.

### 2.6. Microbial Community Analysis Based on 16S rRNA Gene Sequencing

Fecal samples collected from healthy and diarrheic cats were subjected to 16S rRNA gene high-throughput amplicon sequencing, which was performed by Shanghai Meiji Bio-Pharm Technology Co., Ltd., Shanghai, China. The V3–V4 hypervariable region of the bacterial 16S rRNA gene was amplified using the primer pair 338F (5-ACTCCTACGGGAGGCAGCAG-3) and 806R (5-GGACTACHVGGGTWTCTAAT-3). PCR amplicons were sequenced on the Illumina MiSeq platform. Raw sequencing reads were processed via the DADA2 workflow embedded in QIIME2 (version 2022.08) for quality filtering, denoising, chimera removal, and amplicon sequence variant (ASV) generation. Taxonomic annotation of ASVs was conducted against the SILVA database.

A total of six fecal samples from diarrheic cats and six samples from healthy cats were initially enrolled for sequencing. One sample from the healthy group was excluded because the extracted DNA did not meet the quality requirements for library construction. All downstream microbiome analyses were performed based on normalized ASV abundance tables.

Alpha diversity metrics (Chao index), gut microbiome health index (GMHI), and microbial dysbiosis index (MDI) were compared between groups using the Wilcoxon rank-sum test. Beta diversity was visualized by principal component analysis (PCA). Permutational multivariate analysis of variance (PERMANOVA) with 999 permutations based on the Bray–Curtis distance was used to assess the statistical significance of overall microbial community compositional differences between healthy and diarrheic cats. The linear discriminant analysis effect size (LEfSe) algorithm was applied to screen differentially enriched taxa, with the LDA score threshold set to 3.0. The Wilcoxon rank-sum test was used for differential taxon testing at the species level, and multiple comparison correction was performed using the Benjamini–Hochberg false discovery rate (FDR) method. Exact P values were reported in corresponding figure legends where applicable. All microbiome statistical computations were implemented in R software (version 4.2.1; R Core Team, Vienna, Austria, 2022).

### 2.7. Absolute-Quantitative qPCR Targeting the lpxC Gene

Total genomic DNA was extracted from cat fecal samples using the QIAamp PowerFecal Pro DNA Kit (QIAGEN GmbH, Hilden, Germany) following the manufacturer’s instructions. The *lpxC* gene was selected as a molecular marker for Gram-negative bacteria. Primer pairs for *lpxC* are listed in Table 1. To construct the qPCR standard curve, the target *lpxC* amplicon was cloned into the pMD-18T plasmid. The stock recombinant plasmid concentration was determined as 8.44 × 10^10^ copies/μL. Serial 10-fold dilutions of the recombinant plasmid were used to generate the standard curve. The qPCR reaction was performed on a real-time PCR instrument. Absolute *lpxC* gene copy counts were calculated according to the standard curve and normalized to per-gram wet weight of fecal samples. Of the enrolled animals, all 27 diarrheic cats and 7 healthy cats were subjected to *lpxC*-targeted qPCR assay.

### 2.8. Statistical Analysis

All values are presented as the mean ± SEM, and Student’s *t*-test was used to compare differences between groups. For the repeated-measure weight data, we employed Linear Mixed-Effects Models (LMMs) for analysis. The survival functions for each group were estimated using the Kaplan–Meier method. The survival curves were compared for differences between groups using the Log-rank test. All statistical analyses were performed using SPSS 19.0 software, and graphs were generated using GraphPad Prism 9.3 software. A *p*-value of less than 0.05 was considered to indicate a significant difference.

## 3. Results

### 3.1. The Impact of Fmt on Mice Growth Performance and Organ Levels

**Figure 1:**
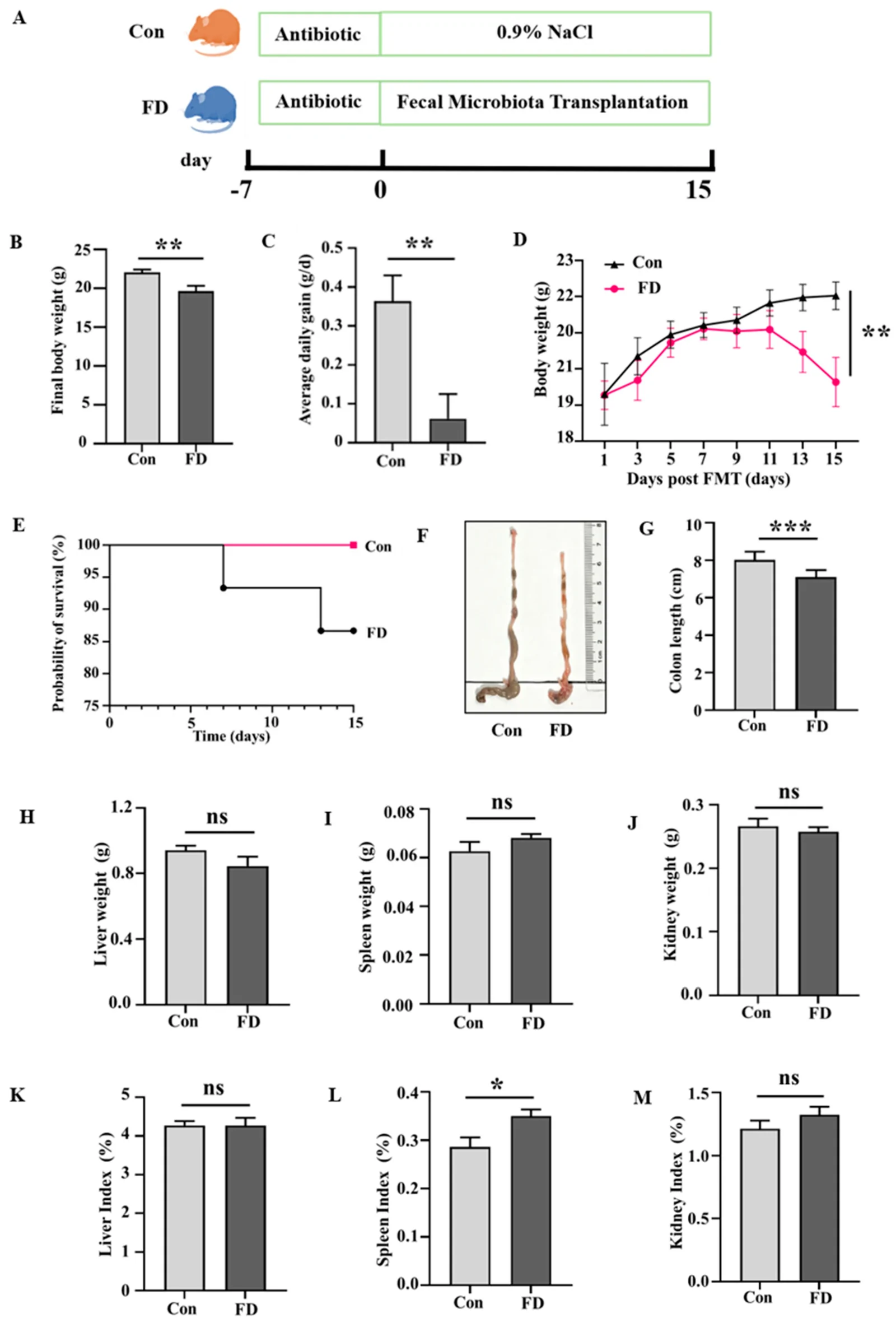
The impact of fecal microbiota transplantation (FMT) on the growth performance and organ indices of mice. (**A**) Experimental design scheme. (**B**) Differences in mouse body weight at the end of the experiment. (**C**) Average daily weight gain. (**D**) Weight changes over fifteen days. (**E**) Mouse survival rate. (**F**) Images of the colon. (**G**) Length of the colon. (**H**–**J**) Weights of the liver, spleen, and kidneys. (**K**–**M**) Liver index, spleen index, and kidney index. Data are presented as mean ± SEM (*n* ≥ 8). * *p* < 0.05, ** *p* < 0.01, *** *p* < 0.001; ns, not significant (*p* ≥ 0.05).

The experimental animal design is shown in Figure 1A. Following gavage of pooled fecal microbes collected from diarrheic cats to antibiotic-treated mice, their final body weight (Figure 1B) and average daily weight gain (Figure 1C) were significantly lower than those of the Con group at the end of the experiment. The body weight of mice in the FD group showed a significant difference from that in the Con group on day 9 (Figure 1D). At the end of the experiment, we found that the microorganism from cats with diarrhea significantly affected the survival rate of the mice (Figure 1E). Subsequently, we observed the colon, and after FMT, the mice’s colonic and cecal contents were not well-filled (Figure 1F), and the length of the colon was significantly reduced (Figure 1G). There were no significant changes in the weights of the liver, spleen, and kidneys (Figure 1H–J). The liver index and kidney index showed no significant change (Figure 1K,M); the spleen index increased significantly (Figure 1L).

### 3.2. The Impact of FMT on the Intestinal Barrier of Mice

**Figure 2:**
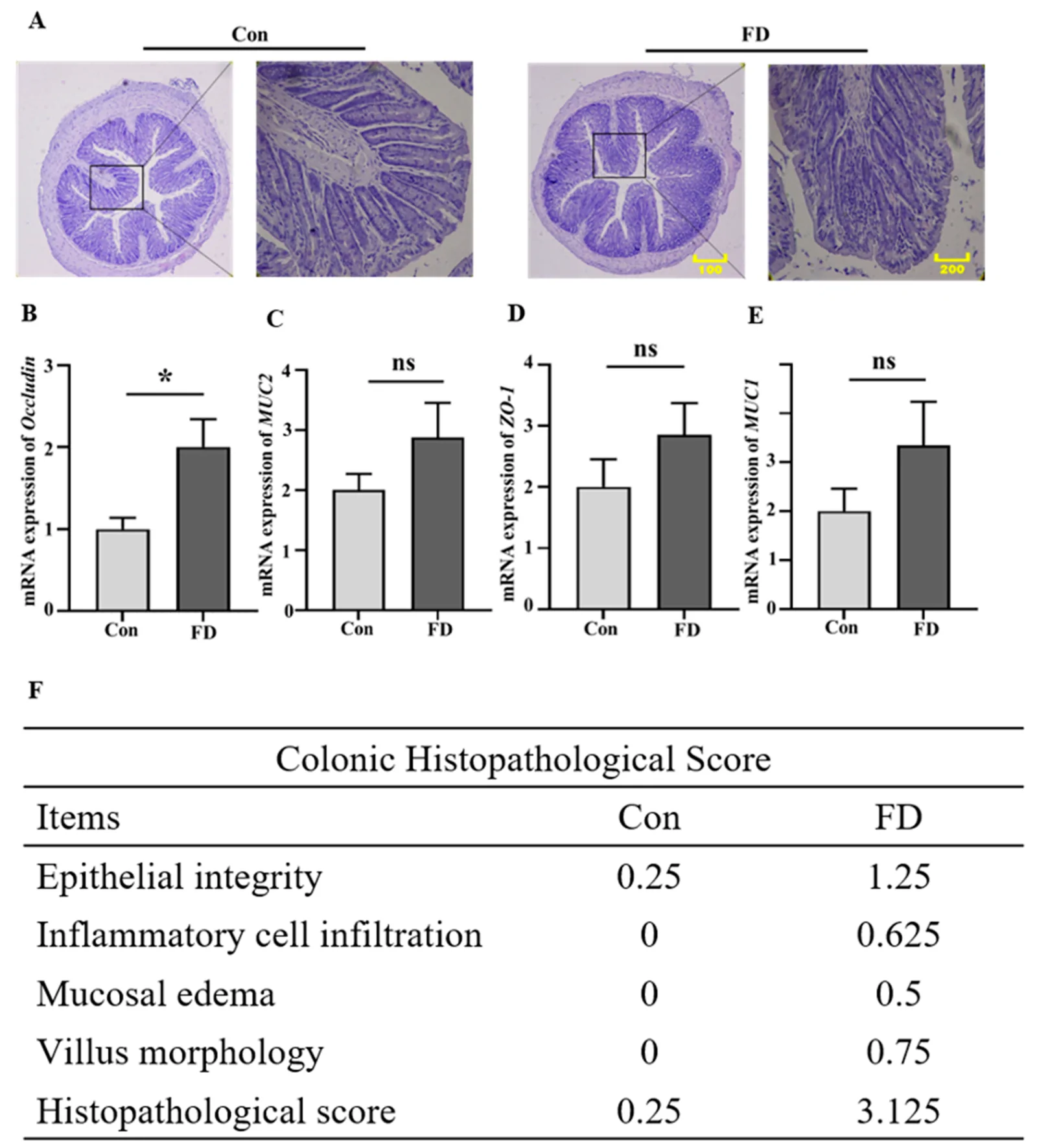
The impact of FMT on the intestinal barrier of the mouse colon. (**A**) H&E staining of the colon. (**B**–**E**) Relative expression levels of the intestinal barrier genes Occludin, ZO-1, MUC1, and MUC2; (**F**) Histopathological score. Data are presented as mean ± SEM (*n* ≥ 8). * *p* < 0.05, ns, not significant (*p* ≥ 0.05).

According to H&E staining, we found obvious lesions in the colon of mice after FMT (Figure 2A). The expression of the tight junction protein Occludin was significantly increased in the FD group, which we interpreted as compensatory upregulation in response to intestinal mucosal injury (Figure 2B), while the expression of *ZO-1*, *MUC1*, and *MUC2* genes increased to varying degrees (Figure 2C–E). Semi-quantitative histological scoring revealed that FD mice exhibited significantly higher intestinal mucosal injury scores compared with the Con group (Figure 2F).

### 3.3. FMT Leads to Macrophage Aggregation and Increased Inflammatory Response in the Colon of Mice

**Figure 3:**
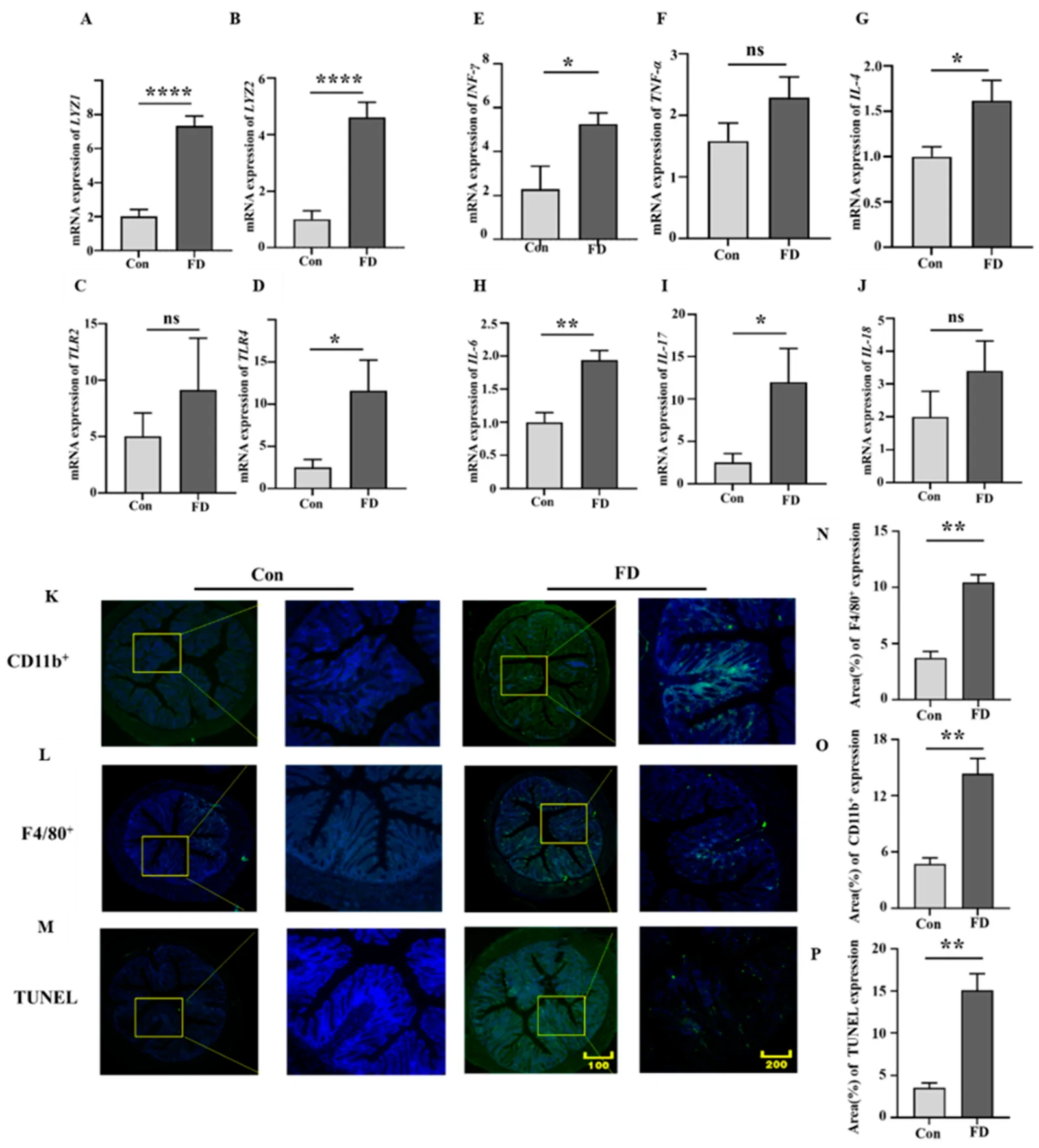
The impact of FMT on macrophage aggregation and inflammatory response in mice. (**A**,**B**) Relative expression levels of the *LYZ1* and *LYZ2* genes; (**C**,**D**) Relative mRNA expression of *TLR2* and *TLR4* in the colon; (**E**–**J**) Expression of inflammation-related genes in the colon; (**K**,**L**) Representative images of CD11b^+^ and F4/80^+^ immunofluorescence in the colon; (**M**) Representative images of TUNEL immunofluorescence in the colon; (**N**) Quantitative analysis of F4/80-positive macrophage infiltration; (**O**) Quantitative analysis of CD11b-positive inflammatory cell accumulation; (**P**) Quantitative analysis of TUNEL-positive apoptotic cells. Data are presented as mean ± SEM (*n* ≥ 8). * *p* < 0.05, ** *p* < 0.01, **** *p* < 0.0001, ns, not significant (*p* ≥ 0.05).

When the body is invaded by bacteria, the number of lysozymes in the intestine increases significantly. The expression of *LYZ1* and *LYZ2* genes in mice after FMT increased significantly (Figure 3A,B). Bacterial infection leads to an increase in the content of lipopolysaccharides (LPS) and Peptidoglycan (PG) in the intestine, and *TLR2* and *TLR4* are the receptors that intestinal cells use to sense LPS and PG. The expression of *TLR2* and *TLR4* genes in the colon of mice after fecal microbiota transplantation increased significantly (Figure 3C,D). Macrophages, upon stimulation by LPS, also aggregate in the colon (Figure 3K,L), and the aggregated macrophages release more inflammatory factors. Therefore, we found that the expression of inflammatory factors in the colon of mice increased to varying degrees (Figure 3E–J). Inflammatory factors, in turn, promote apoptosis, and the number of apoptotic cells in the colon of mice after FMT increased significantly (Figure 3M). The area percentages of F4/80^+^ macrophages, CD11b^+^ inflammatory cells and TUNEL apoptotic cells were markedly higher in the FD group compared with the Con group (Figure 3N–P).

### 3.4. Differences in Microbiota Between the Feces of Healthy Cats and Cats with Diarrhea

**Figure 4:**
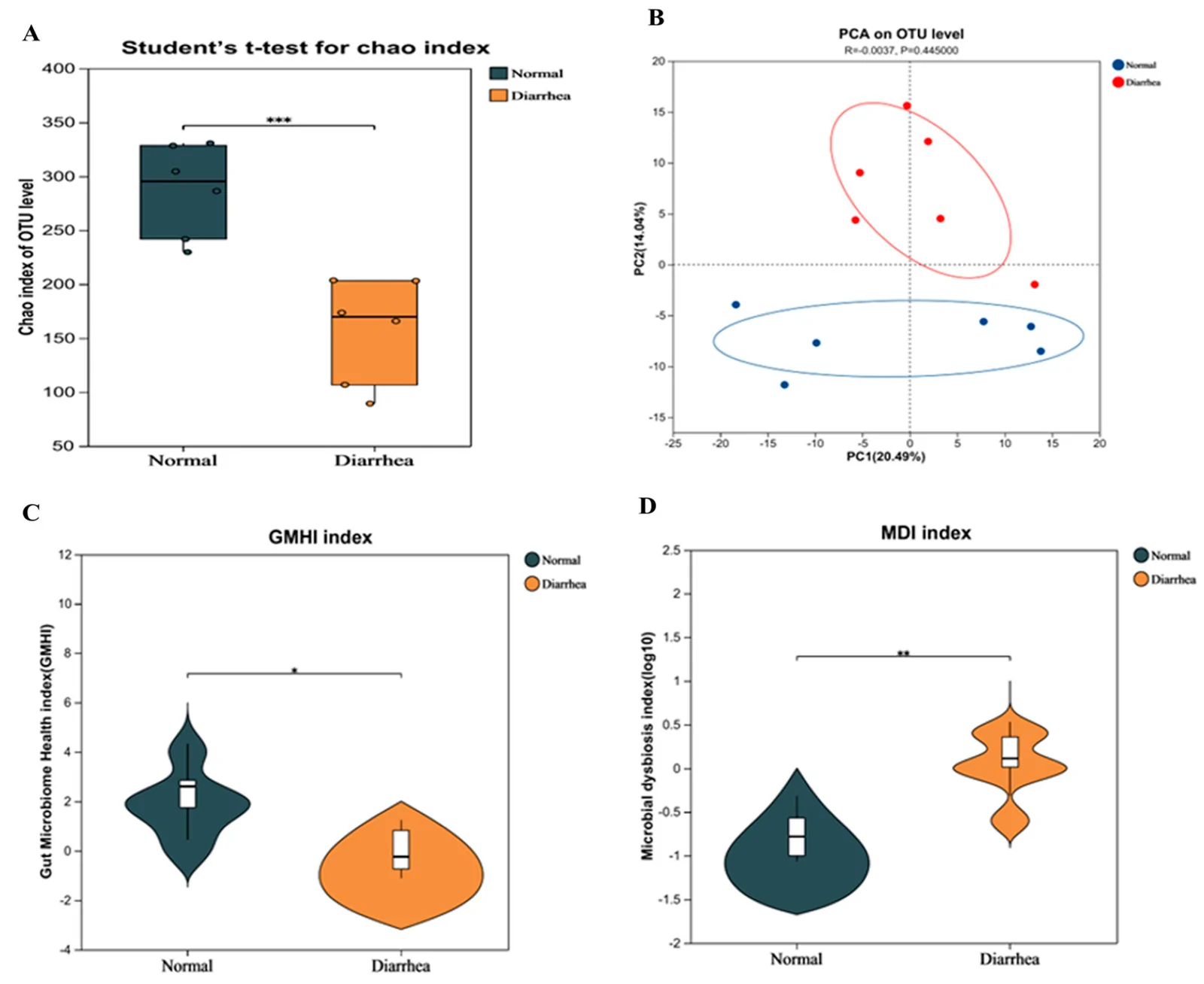
Differences in fecal microbiota between healthy cats and cats with diarrhea. (**A**) Chao index. (**B**) Principal component analysis (PCA) plot of gut microbiota at the OTU level. (**C**) Health index (GMHI) of gut microbiota at the OTU level. (**D**) Microbial dysbiosis index (MDI) of gut microbiota at the OTU level. Data are presented as mean ± SEM (*n* = 6 per group for 16S rRNA gene sequencing). * *p* < 0.05, ** *p* < 0.01, *** *p* < 0.001.

**Figure 5:**
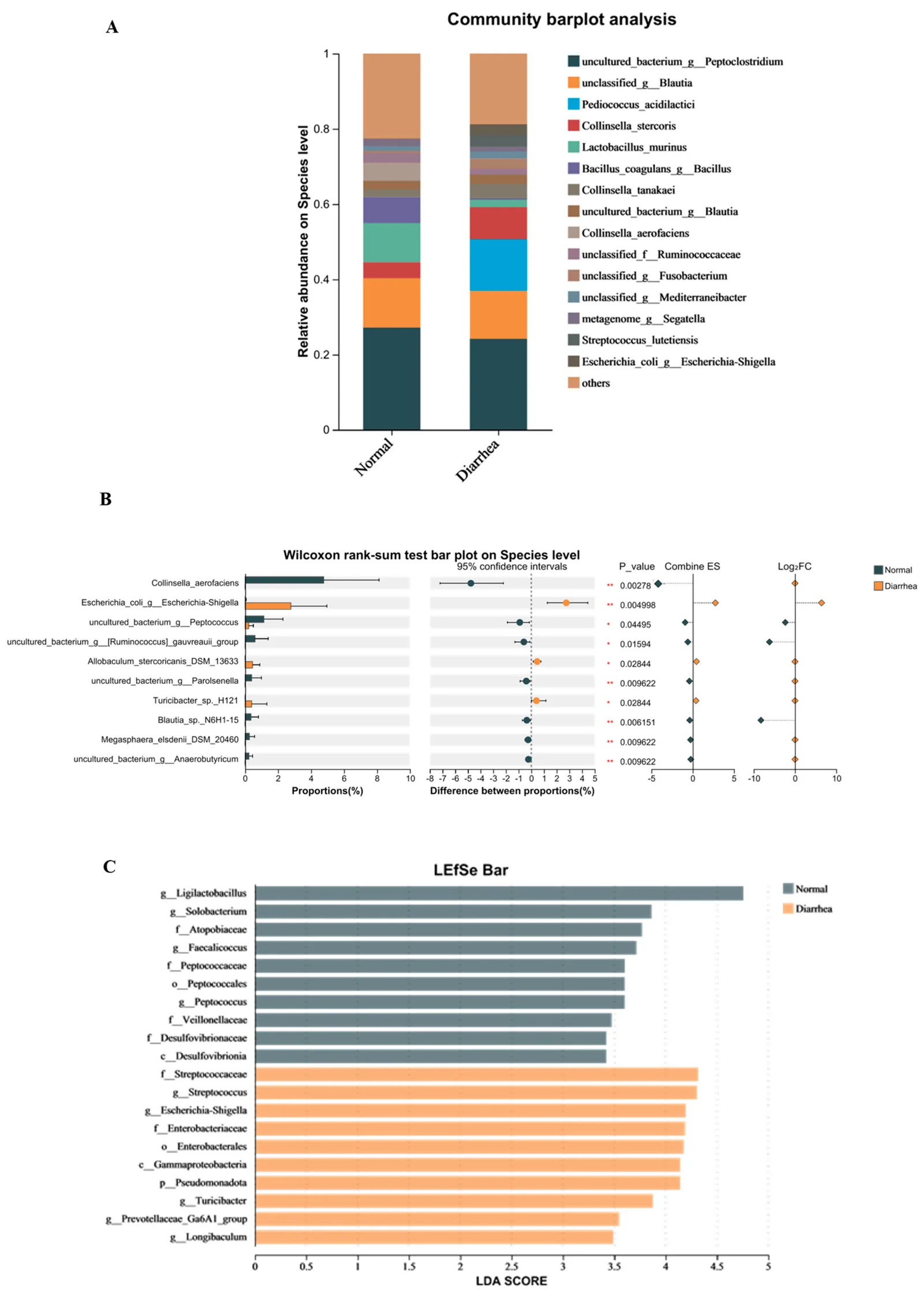
(**A**) Bar chart showing differences in gut microbiota at the species level; (**B**) Linear discr minant effect size (LEfSe) analysis bar chart of differential microbial communities; (**C**) Wilcoxon rank-sum test results of differential microbial communities at the species level. Data are presented as mean ± SEM (*n* = 6 per group for 16S rRNA gene sequencing). * *p* < 0.05, ** *p* < 0.01.

The Chao index is mainly used to assess the richness of items in a sample. The Chao index of microbial flora in the feces of healthy cats was significantly higher than that in the feces of cats with diarrhea (Figure 4A). In principal component analysis (PCA), the community structure of the fecal microbiota of healthy cats and cats with diarrhea was significantly different (Figure 4B). The health index (GMHI) of the gut microbiota of healthy cats was significantly higher than that of cats with diarrhea (Figure 4C), while the microbial dysbiosis index (MDI) showed the opposite trend (Figure 4D). At the species level, the gut microbiota of both healthy cats and cats with diarrhea were mainly dominated by *Peptoclostridium* and *Blautia*, with *Lactobacillus murinus* and *Bacillus coagulans* also playing a major role in the gut of healthy cats, while *Collinsella stercoris* and *Escherichia*–*Shigella* played a major role in the gut of cats with diarrhea (Figure 5A). In LEfSe analysis, *Ligilactobacillus* played a major role in healthy cats, while *Streptococcaceae*, *Streptococcus*, and *Escherichia*–*Shigella* played a major role in cats with diarrhea (Figure 5B). The Wilcoxon rank sum test showed that the content of *Escherichia*–*Shigella* in feces of cats with diarrhea was significantly higher than that of healthy cats (Figure 5C). We can infer that the microbes in the gut of healthy cats may enhance intestinal resistance due to the high content of lactic acid bacteria, while the increased content of *Escherichia*–*Shigella* in the gut of cats with diarrhea may directly or indirectly damage the intestinal health of cats.

### 3.5. Detection Rate of Pathogenic Bacteria in Feces

**Figure 6:**
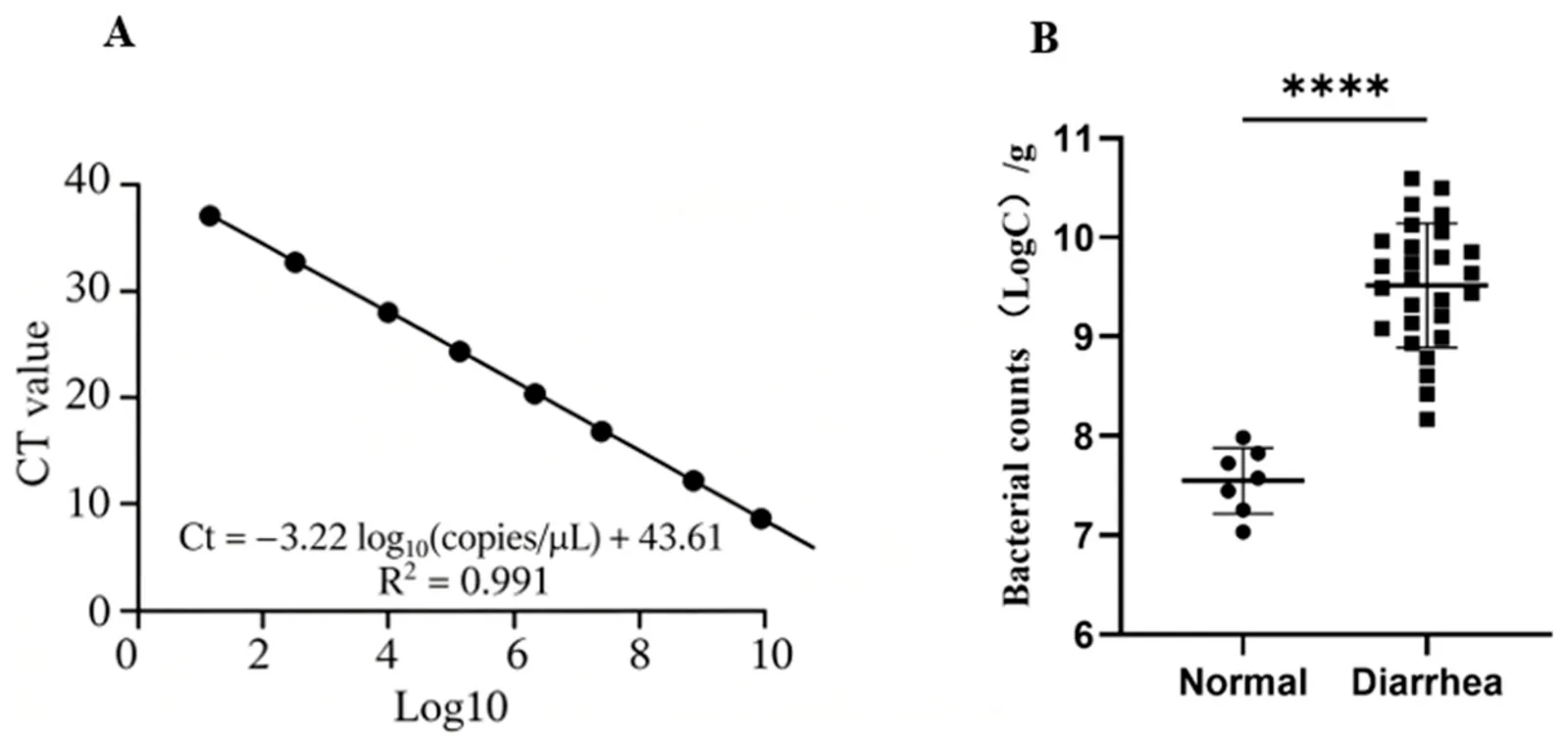
Absolute quantification of *IpxC* gene copies in fecal samples from healthy and diarrheic cats. (**A**) Standard curve of qPCR amplification targeting the *IpxC* gene; (**B**) Comparison of *IpxC* bacterial loads (log_10_ copies/g feces) between healthy cats (Normal, *n* = 7) and diarrheic cats (Diarrhea, *n* = 27). **** *p* < 0.0001.

Subsequently, absolute quantification of fecal bacteria carrying the *IpxC* gene was performed via qPCR in healthy and diarrheic cats. The standard curve for *IpxC* qPCR amplification presented the formula Ct = −3.22 log_10_ (copies/Μl) + 43.61 with an R^2^ value of 0.991, confirming robust linearity and satisfactory quantification performance of this detection system (Figure 6A). After normalization to per gram of feces, log_10_-transformed bacterial loads of the *IpxC* gene were compared between groups. Fecal *IpxC* bacterial abundance was markedly elevated in diarrheic cats (*n* = 27) relative to healthy individuals (*n* = 7), with an extremely significant statistical difference (*p* < 0.0001). These data demonstrated that overcolonization of *IpxC*-harboring bacteria is strongly associated with feline diarrhea (Figure 6B).

## 4. Discussion

Fecal microbiota transplantation (FMT) is an innovative therapeutic approach that entails transferring the gut microbiota from a healthy donor to a recipient in order to restore microbial equilibrium. Over recent years, FMT has demonstrated considerable promise in addressing a spectrum of medical conditions. Notably, in cases of recurrent Clostridium difficile infections, FMT has achieved cure rates of up to 90%, markedly surpassing the efficacy of vancomycin. Additionally, FMT has proven beneficial in managing inflammatory bowel disease (IBD) and irritable bowel syndrome (IBS), with a notable increase in *Faecalibacterium prausnitzii* observed within the recipient’s gut following the procedure [18,19,20]. FMT can effectively transfer microbes from the donor to the recipient. Cats have a digestive structure that is quite different from that of omnivorous animals like dogs. As obligate carnivores, cats have a gut structure and microbiome that are uniquely adapted to their high-protein, high-fat dietary needs. This adaptation results in a considerably lower ratio of intestinal length to body length in cats compared with omnivores, with a less developed cecum and a comparatively simple microbial community in the colon [21]. Therefore, we infer that feline intestinal health issues may not be directly related to the microbes in their gut, which has led to less focus on changes in the feline gut microbiome. However, in domestic settings, cats are frequently treated with deworming medications and vaccinations, which primarily safeguard against parasitic and viral infections and offer minimal protection against bacterial infections. Although the gut microbial community in cats is relatively straightforward, the impact of microbial structural changes on feline intestinal health remains to be fully understood [13,22]. In this experiment, microbes from the feces of cats with diarrhea were transplanted into antibiotic-treated mice, and it was found that the mice’s body weight significantly decreased, and the splenic index increased. Bacterial infections in mice typically lead to a substantial decrease in body weight, and the spleen, being the body’s largest secondary lymphoid organ and central to immune responses, tends to enlarge noticeably as a result of immune cell proliferation [7,23,24]. Similar phenomena were observed in the mice after FMT, proving that the gut of cats with diarrhea contains certain pathogenic bacteria that cause pathological changes in mice.

The cecum and colon serve as the primary habitats for microbial aggregation. Upon bacterial infection, the colon’s length is typically shortened, its contents diminished, and the intestinal barrier compromised [25]. In our experiment, we observed analogous alterations in the mice, accompanied by signs of inflammatory infiltration within their colons. One potential hypothesis is that presumed intestinal barrier impairment may prompt up-regulation of genes related to intestinal integrity, possibly representing an adaptive response attempting to repair bacteria-induced damage. Notably, *Occludin* expression was significantly elevated in FD mice. Increased *Occludin* expression usually represents strengthened intestinal barrier function under homeostatic conditions. However, combined with H&E histopathological evidence of mucosal injury and inflammatory cell infiltration, this elevation was considered a compensatory response of intestinal epithelial cells against inflammatory damage. Upon inflammatory stimulation, epithelial cells upregulate tight junction proteins to mitigate disruption of intestinal barrier integrity, which is a well-documented compensatory mechanism during gut inflammation. Lysozyme is mainly secreted by Paneth cells in the gut and is an important antimicrobial peptide. It plays a pivotal role in augmenting the gut’s defenses and preserving microbial equilibrium when faced with overgrowth of certain pathogenic bacteria [26,27]. Post-FMT, there was a marked escalation in lysozyme expression within the colonic tissue of the mice, suggesting that the fecal matter of cats with diarrhea is teeming with pathogenic bacteria. These bacteria not only undermine the colon’s health but also trigger an increase in lysozyme production, indicative of the body’s struggle to combat the microbial onslaught.

*TLR4* mainly recognizes LPS from Gram-negative bacteria, and *TLR2* mainly recognizes components such as peptidoglycan from Gram-positive bacteria, thereby initiating an immune response against pathogenic bacteria. These recognition events trigger an immune response against pathogenic bacteria. In prior studies, invasion of the body by pathogenic bacteria led to the activation of MyD88-dependent signaling cascades, including NF-κB and MAPK pathways, within the gut-associated TLR4 and TLR2 receptors upon their binding to LPS and peptidoglycan. This activation process fosters inflammatory responses [25,28,29]. In our experiment, the expression levels of both *TLR4* and *TLR2* were observed to increase, with *TLR4* showing a particularly significant rise. These receptors also serve as principal sensors for macrophages. Once there, macrophages, stimulated by LPS, differentiate into M1-type inflammatory cells that further release pro-inflammatory mediators. These mediators not only initiate defensive mechanisms but also recruit other immune cells, facilitating the swift assembly of neutrophils and their subsequent removal through apoptosis [30,31,32,33]. In this experiment, macrophages in the colon significantly aggregated, inflammatory factors were elevated to varying degrees, and the aggregation of apoptotic proteins also significantly increased. LPS is the main component of the outer membrane of Gram-negative bacteria and is a potent immune activator. TLR4 is the main recognition receptor for LPS, indicating that the main pathogenic bacteria in the feces of cats are Gram-negative bacteria, which are the main cause of infection in mice. Increased TLR4 expression and inflammatory responses observed in FMT-treated mice are consistent with exposure to LPS. One plausible hypothesis is that LPS derived from Gram-negative bacteria may trigger intestinal inflammation and barrier injury through TLR4 signaling. The observed macrophage aggregation, elevated inflammatory cytokines and increased apoptotic protein abundance in FMT mice collectively indicated that LPS-carrying Gram-negative bacteria function as the dominant pathogenic agent triggering intestinal inflammation.

Based on the above FMT mouse mechanistic findings, we hypothesized that excessive proliferation of intestinal Gram-negative bacteria contributes to feline diarrhea. To quantify total Gram-negative bacterial load objectively, absolute qPCR targeting the Gram-negative-specific marker gene *IpxC* was performed on feline fecal samples. *IpxC* is an evolutionarily conserved essential gene widespread in Gram-negative bacteria, encoding the key enzyme for LPS/Lipid A biosynthesis and serving as a reliable molecular marker to quantify total loads of Gram-negative bacteria via qPCR. Quantitative results demonstrate that log_10_-transformed *IpxC* bacterial copies per gram of feces were extremely higher in diarrheic cats compared with healthy controls, solidifying the correlation between Gram-negative bacterial overgrowth and feline diarrhea. It is well established that *Shigella* invades colonic epithelium, destroys intestinal barrier integrity, induces mucosal inflammation, triggers macrophage pyroptosis and facilitates IL-1β release [34,35,36,37]. These pathological mechanisms perfectly match the inflammatory, barrier-damaged and pyroptosis phenotypes detected in FMT recipient mice [13,38]. Combining in vivo animal transplantation, molecular immune detection and fecal absolute quantification, we speculate that overcolonization of Gram-negative bacteria represented by *Escherichia*–*Shigella* may be one potential contributing factor for diarrhea in the enrolled cats. The relatively simple gut microbiota of cats does not mean bacteria are irrelevant to intestinal disease; instead, abnormal proliferation of individual pathogenic Gram-negative taxa might readily disrupt intestinal homeostasis and elicit diarrheal symptoms. Although FMT using fecal microbiota from diarrheic cats induced intestinal inflammatory injury and disruption of intestinal barrier function in recipient mice, we did not characterize the fecal microbiota of mice before and after FMT or perform longitudinal quantification of *Escherichia*–*Shigella*. Thus, we lack direct evidence to confirm successful engraftment of donor-origin *Escherichia*–*Shigella*. The observed mucosal injury may be attributed to transferred microbial metabolites, bacterial structural components, or the combined effects of the entire donor microbial community, instead of stable colonization of *Escherichia*–*Shigella*.

This study has several limitations that need to be addressed in future experiments. In terms of design, there is a lack of a control group for healthy cats undergoing FMT, an antibiotic-only treatment group, and an experimental group for stress isolation. This makes it impossible to distinguish the independent contributions of the FMT effect from those of antibiotics and operational stress. At the microbiological level, there was no detection of colonization by *Escherichia*–*Shigella* bacteria in the feces of mice after FMT. This prevents the elucidation of the relationship between the changes in the intestinal barrier and the occurrence of inflammation and the *Escherichia*–*Shigella* bacteria. It can only be inferred that these changes in the mice are related to the *Escherichia*–*Shigella* bacteria based on the differences in intestinal microorganisms between normal cats and cats with diarrhea.

## 5. Conclusions

In summary, our research results indicate that although the gut microbiota structure in cats is relatively simple, changes in microbial composition can also contribute to feline diarrhea. Fecal microbiota from diarrheic cats can disrupt intestinal barrier homeostasis, elevate pro-inflammatory mediators and trigger macrophage aggregation in antibiotic-treated recipient mice. Among differentially enriched pathogens, *Escherichia*–*Shigella* is markedly enriched in diarrheic cat feces and may represent a relevant candidate pathogen. While *Escherichia*–*Shigella* may not serve as the direct primary cause of feline diarrhea in natural cases, it is a zoonotic pathogen that deserves attention for potential cross-species transmission risks.

## Figures and Tables

**Table 1:** Primer design and synthesis.

Item.	Gene		Sequence (5′ → 3′)
1	*Occludin*	F	CTTCGACTCGCTGCTGAATC
		R	ACCTCATCGTCTTCCATGCA
2	*ZO-1*	F	GCCCACCAAGATCGTCTACT
		R	GTAGTCCTTGCGGTCGTAGG
3	*Muc1*	F	ATGCCCTTGCGTCCATAACA
		R	AGGAGCAGTGTCCGTCAAAG
4	*Muc2*	F	TGGAACCACGATGACAGCCT
		R	TTCCCGAATTCCATGGGTGT
5	*LYZ1*	F	AGAGCTGCCCCTTTCATCTT
		R	ATGCCTCATGACACTGGGAA
6	*LYZ2*	F	GCACAGGAGTCTCAGTGGAT
		R	AGAAAGACAGGTCACGGGTT
7	*TLR2*	F	CTGAGAATGATGTGGGCGTG
		R	TTAAAGGGCGGGTCAGAGTT
8	*TLR4*	F	GGAACTTTGTCCCTGGCAAG
		R	TGGGGAGACAGCACAAAGAT
9	*INFγ*	F	GTCAACAACCCACAGGTCCA
		R	ACTCCTTTTCCGCTTCCTGA
10	*TNFα*	F	CCACCACGCTCTTCTGTCTA
		R	GGTCTGGGCCATAGAACTGA
11	*IL-4*	F	ACTTGAGAGAGATCATCGGCA
		R	AGCTCCATGAGAACACTAGAGTT
12	*IL-6*	F	CTGCAAGAGACTTCCATCCAG
		R	AGTGGTATAGACAGGTCTGTTGG
13	*IL-17*	F	ACCTGGAACTGAATGCCTGA
		R	GTCCCTCGATGTGGCTACTT
14	*IL-18*	F	CCCAATGAGTAGGCTGGAGA
		R	TCTGGACCCATTCCTTCTTG
15	*β-actin*	F	GTGACGTTGACATCCGTAAAGA
		R	GCCGGACTCATCGTACTGAG
16	*IpxC*	F	ATCGTCGACCTGGTGGATGA
		R	GCGATGATGTTGGTGGTGTT

## Data Availability

The data presented in this study are openly available in [science data bank] at [https://doi.org/10.57760/sciencedb.32412].

## Supplementary Materials

The following supporting information can be downloaded at: https://www.mdpi.com/article/10.3390/vetsci13090976/s1, Supplementary Table S1: Basic clinical information of enrolled cats.
